# Egg consumption and risk of type 2 diabetes: a prospective study and dose–response meta-analysis

**DOI:** 10.1007/s00125-016-3923-6

**Published:** 2016-03-18

**Authors:** Alice Wallin, Nita G. Forouhi, Alicja Wolk, Susanna C. Larsson

**Affiliations:** Unit of Nutritional Epidemiology, Institute of Environmental Medicine, Karolinska Institutet, Box 210, 171 77 Stockholm, Sweden; Medical Research Council Epidemiology Unit, University of Cambridge School of Clinical Medicine, Institute of Metabolic Science, Cambridge, UK

**Keywords:** Cohort study, Diabetes, Eggs, Meta-analysis, Prospective study

## Abstract

**Aims/hypothesis:**

In this study, we aimed to investigate the association between egg consumption and type 2 diabetes risk in the Cohort of Swedish Men and to conduct a meta-analysis to summarise available prospective evidence on this association.

**Methods:**

We followed 39,610 men (aged 45–79 years) from 1998 up to 2012 for incident type 2 diabetes. Egg consumption was assessed at baseline using a food frequency questionnaire. HRs (95% CIs) were estimated using Cox proportional hazards regression models. We searched PubMed (up to 14 December 2015) and reference lists of retrieved articles to identify eligible studies for meta-analysis.

**Results:**

During the 15 years of follow up, 4,173 men were diagnosed with type 2 diabetes. Compared with men who consumed eggs <1 time/week, the multivariable-adjusted HRs were 0.98 (95% CI 0.92, 1.05), 1.11 (95% CI 0.99, 1.24) and 1.11 (95% CI 0.95, 1.29) for egg consumption 1–2, 3–4 and ≥5 times/week, respectively (*p*_trend_ = 0.06). In a random-effects dose–response meta-analysis, heterogeneity in the overall estimate was partly explained by differences across regions. The overall HRs for type 2 diabetes for each 3 times/week increment in consumption were 1.18 (95% CI 1.13, 1.24) in five US studies (*I*^2^ = 0%) and 0.97 (95% CI 0.90, 1.05) in seven non-US studies.

**Conclusions/interpretation:**

Our findings in Swedish men do not support an association between egg consumption and risk of type 2 diabetes. In a meta-analysis, frequent egg consumption was associated with a higher risk of type 2 diabetes in US studies only. Egg consumption habits and associated overall dietary patterns may differ between populations and could potentially explain the discrepancies between reported results. Given the inconsistent results, this relationship warrants further study.

## Introduction

Type 2 diabetes is a major global public health issue, and the rapid increase in prevalence over the past decades is expected to continue [1]. Identifying modifiable factors, such as dietary components, that may influence the risk of type 2 diabetes could be important for reducing the disease burden. Dietary factors such as egg consumption may influence the risk of developing type 2 diabetes. Eggs are rich in dietary cholesterol and protein, and prospective studies have found positive associations of cholesterol [2, 3] and protein [4, 5] intake with type 2 diabetes risk. Egg is also a rich source of many potentially beneficial dietary components such as vitamins, minerals and carotenoids.

Epidemiological evidence for an association between egg consumption and risk of type 2 diabetes from prospective [6–15], case–control [16] and cross-sectional studies [17] is inconsistent. In two previous meta-analyses, including prospective data from US studies only, egg consumption was associated with a higher risk of type 2 diabetes [18, 19]. However, several recent studies in European [9, 10], Asian [11] and US [12] populations have reported no association or an inverse association [13]. We therefore aimed to assess the association between egg consumption and risk of type 2 diabetes in the prospective Cohort of Swedish Men (COSM). We also conducted an updated meta-analysis of prospective studies to reassess previous results.

## Methods

### Study population

The COSM is a prospective population-based study of men from the Örebro and Västmanland counties of central Sweden. Recruitment started in late 1997 when all men resident in these counties who were born between 1918 and 1952 received a questionnaire on diet and other lifestyle factors: 48,850 men (49%) responded. An extended questionnaire on health, including a question on diabetes status, was distributed in 2008 (response rate 70%). We excluded men who provided an incorrect or an incomplete national identification number (*n* = 205), returned a blank questionnaire (*n* = 92), or were diagnosed with cancer (other than non-melanoma skin cancer; *n* = 2,592) or died (*n* = 55) before 1 January 1998. In addition, we excluded men with prevalent diabetes as determined via registry data and baseline self-reports (*n* = 3,404), men who reported a diabetes diagnosis in the 2008 questionnaire that could not be confirmed by registry data (*n* = 67) and men who were registered with diabetes other than type 2 during the follow-up period (*n* = 200). After further exclusion of men with implausible energy intake (±3 SD from the log-transformed mean energy intake; *n* = 485) and those with no information on egg consumption (*n* = 2,140), 39,610 men were included in the analysis. The study was approved by the Regional Ethical Review Board at Karolinska Institutet, Stockholm, Sweden. A returned completed questionnaire was considered to imply informed consent.

### Assessment of diet and other covariates

A 96-item food frequency questionnaire (FFQ) on the average food consumption during the past year was used to assess baseline diet (http://ki.se/en/imm/cosm-a-cohort-of-50000-swedish-men). For egg consumption, participants could choose from eight predefined frequency categories (<1 time/month; 1–3 times/month; 1–2 times/week; 3–4 times/week; 5–6 times/week; 1 time/day; 2 times/day; ≥3 times per day). Intake of nutrients and total energy was calculated based on age-specific (≤52, 53–61, 62–69 and ≥70 years) portion sizes derived from two 1-week weighted dietary records completed by a random sample of 152 men and on food composition values obtained from the Swedish National Food Agency Database [20]. Nutrient intake values were adjusted to the mean energy intake in the cohort using the residual method [21]. In a validation study in Swedish men, Spearman’s *r* coefficients between FFQ-based estimates and the mean of 14 repeated 24-h recall interviews were 0.65 for macronutrients and 0.62 for micronutrients [22]. Specific food items were validated in a sample of 129 women of the same age from the same study area. For egg consumption, Spearman’s *r* coefficient between the FFQ and the average of four 1-week weighted diet records was 0.5 (A. Wolk, unpublished data).

The baseline questionnaire further included questions on height, body weight, education, alcohol consumption, smoking habits and physical activity. BMI was calculated by dividing the weight (in kg) by the square of the height (in m). Physical activity was assessed via questions on active time spent at work, home/housework, walking/bicycling and exercise, as well as inactive time spent watching TV/reading and sleeping. The reported time per day spent on these activities was multiplied by their typical energy expenditure requirement expressed in metabolic equivalents (METs), and then combined to create a MET-h/day score [23]. History of cardiovascular disease (CVD; myocardial infarction, angina and stroke) at baseline was identified by linkage with the Swedish National Patient Register.

### Case ascertainment and follow-up

Incident cases of type 2 diabetes that occurred between 1 January 1998 and 31 December 2012 were identified by linkage of the study cohort with the Swedish National Diabetes Register and the Swedish National Patient Register. The Diabetes Register contains information from regular patient visits and retrospective recording of diabetes onset year. The coverage is estimated to be 90–100% in the study area [24]. ICD-10, code E11 was used to identify type 2 diabetes cases in the cohort from the National Patient Register. Reporting to the National Patient Register is mandatory; it includes information on main and secondary diagnoses for all inpatient care visits in Sweden since 1987 and all outpatient visits from private and public caregivers since 2001. The first recorded date in either of the two registers was considered the diagnosis date. The dates of all deaths were obtained from the Swedish Death Register.

### Statistical analysis of the cohort data

For all participants, person-time was measured from 1 January 1998 until the date of type 2 diabetes diagnosis, date of death or the end of follow-up, whichever came first. We used Cox proportional hazards models with age as the underlying timescale to estimate age- and multivariable-adjusted HRs (and corresponding 95% CIs) for the association between egg consumption and incidence of type 2 diabetes. We categorised egg consumption as <1, 1–2, 3–4 and ≥5 times/week. As there were only a few non-consumers of eggs in the study population, the first two FFQ categories (<1 and 1–3 times/month) were combined. Likewise, because few participants consumed more than 5–6 times/week, the highest four FFQ categories (5–6 times/week, 1 time/day, 2 times/day and 3 times/day) were combined into a single group. Trends across categories were tested by modelling the median value of each exposure category as a continuous variable. All multivariable models included BMI (<20, 20–24.9, 25–29.9 or ≥30 kg/m^2^), physical activity (MET-h/day; quartiles), education (primary school, high school or university), cigarette smoking (never, former, current ≤10 cigarettes/day or >10 cigarettes/day), total energy intake (kJ/day; quartiles), alcohol intake (g/day; quartiles) and baseline history of CVD (yes/no). In a second multivariable model, we further adjusted for dietary factors, including coffee consumption (cups/day; quartiles) and intake of red meat, processed meat, fish, fruit, vegetables, white bread, caviar, sweet buns/biscuits and fibre (g/day; quartiles). We also considered the consumption of tea, orange/grapefruit juice, sweetened beverages and French fries: these variables did not influence the estimates and were not included in the final model. A separate missing category was used to handle missing covariate data (<2% was missing, with the exception of specific physical activity variables [<10%]). No evidence of violation of the proportional hazards assumption was observed using the Schoenfeld residuals test.

Stata 13.0 (StataCorp, College Station, TX, USA) was used for the statistical analyses, and statistical significance was set at *p* < 0.05.

### Meta-analysis

We conducted a meta-analysis that included findings from the COSM and previous prospective studies that reported risk estimates for type 2 diabetes by egg consumption. Studies were identified by searching PubMed from 1966 until 14 December 2015 using the search term ‘egg’ combined with ‘diabetes’. No language or other restrictions were imposed. We also reviewed the reference lists of identified articles to find further relevant studies. From each study, we extracted the most fully adjusted risk estimates, except when adjustment was made for dietary cholesterol intake. Literature searching and data extraction were performed by two investigators (A. Wallin and S.C. Larsson), and any disagreements were resolved by consensus. We conducted a random-effects dose–response meta-analysis using methods reported in a previous meta-analysis [25]. For each study, we estimated a HR for each 3 times/week increment in egg consumption. If egg consumption was reported in grams, we converted the intake into servings by assuming that one medium egg weighs 55 g. Statistical heterogeneity was evaluated with the *Q* and *I*^2^ statistics [26], and potential publication bias was assessed with the Egger’s test [27]. Potential sources of heterogeneity were assessed by meta-regression and stratified analyses by sex, region (US and non-US studies), country (for non-US studies: Sweden, Finland, and other), follow-up (<15 and ≥15 years) and date of baseline data collection (before or after 1990). We used Stata to analyse the data.

## Results

### The COSM

During 15 years of follow up (510,365 person-years), 4,173 men were diagnosed with type 2 diabetes. At baseline, the mean ± SD egg intake in the cohort was 1.4 ± 1.5 times/week. Men with higher egg consumption were more likely to be current smokers and had on average a higher intake of alcohol and a higher consumption of other foods and food groups (Table 1).

**Table 1:** Age-standardised baseline characteristics of 39,610 participants of the COSM, by categories of egg consumption

Characteristic	Egg consumption, times/week (median)			
	<1 (0.5)	1–2 (1.5)	3–4 (3.5)	≥5 (7)
Participants, *n*	18,731	16,249	3,102	1,528
Age (mean ± SD), years	60.0 ± 9.6	60.1 ± 9.6	60.9 ± 9.4	61.6 ± 9.5
BMI (mean), kg/m^2^	26	26	26	26
Total physical activity (mean), MET-h/day	41	42	42	42
University education, %	16	18	17	16
Current smokers, %	24	24	29	33
Alcohol (mean), g/day	14	16	19	22
Energy intake (mean), kJ/day	10,600	11,600	12,200	13,500
History of CVD, %	11	10	8	9
Food intake				
Coffee (mean), cups/day	3.5	3.5	3.6	3.8
Red meat (mean), g/day	55	62	67	85
Processed meat (mean), g/day	36	43	48	63
Fish (mean), g/day	29	33	36	50
Fruit (mean), g/day	170	190	200	210
Vegetables (mean), g/day	130	140	150	170
White bread (mean), g/day	93	96	101	107
Caviar^a^ (mean), g/day	2.3	3.2	4.4	7.2
Sweet buns/biscuits (mean), g/day	22	23	23	25
Fibre^b^ (mean), g/day	31	30	29	28
Dietary cholesterol^b^ (mean), mg/day	310	360	440	580
Dietary protein^b^ (mean), g/day	100	100	100	110

All variables except age are standardised to the age distribution of the study cohort

^a^Including Swedish sandwich caviar, a processed bread spread made from sugar, salted cod and/or saithe roe that is usually smoked and often consumed with eggs

^b^Adjusted to the mean energy intake in the cohort

Whereas a direct association was observed in the age-adjusted model and after multivariable adjustment for BMI, physical activity, education, smoking, total energy intake, alcohol intake and history of CVD, no statistically significant association remained after additional adjustment for dietary factors (Table 2). Compared with men who consumed eggs <1 time/week, the HR for men who consumed eggs ≥5 times/week was 1.11 (95% CI 0.95, 1.29). Further adjustment for dietary cholesterol (mg/day; quartiles) and protein intake (g/day; quartiles) did not have a marked impact on the observed association. The HRs of type 2 diabetes for egg consumption ≥5 times/week vs <1 time/week were 1.14 (95% CI 0.97, 1.36) after adjustment for dietary cholesterol intake and 1.10 (95% CI 0.95, 1.28) after adjustment for protein intake.

**Table 2:** HRs (95% CI) for type 2 diabetes by egg consumption in the COSM, 1998–2012

Statistic	Egg consumption, times/week (median)				*p* for trend
	<1 (0.5)	1–2 (1.5)	3–4 (3.5)	≥5 (7)	
Cases (*n*)	1,909	1,669	391	204	
Person-years	241,647	210,625	39,312	18,781	
Age-adjusted model	1.00	1.00 (0.94, 1.07)	1.23 (1.10, 1.37)	1.33 (1.14, 1.53)	<0.001
Multivariable model 1^a^	1.00	1.01 (0.95, 1.08)	1.17 (1.05, 1.31)	1.19 (1.03, 1.38)	0.002
Multivariable model 2^b^	1.00	0.98 (0.92, 1.05)	1.11 (0.99, 1.24)	1.11 (0.95, 1.29)	0.06

^a^Adjusted for age (timescale), BMI (<20, 20–24.9, 25–29.9 or ≥30 kg/m^2^), physical activity (MET-h/day; quartiles), education (primary school, high school, university), cigarette smoking (never, former, current: ≤10 cigarettes/day or >10 cigarettes/day), total energy intake (kJ/day; quartiles), intake of alcohol (g/day; quartiles) and history of cardiovascular disease at baseline (yes/no)

^b^Additionally adjusted for coffee consumption (cups/day; quartiles) and intakes of red meat, processed meat, fish, fruit, vegetables, white bread, caviar, sweet buns/biscuits and fibre (g/day; quartiles)

### Meta-analysis

We performed a meta-analysis by combining the results of the Swedish cohort with the findings of previous prospective studies. Our literature search identified 601 articles, of which seven reported the results of prospective studies on egg consumption and risk of type 2 diabetes [7–13]. One article reported results from two separate cohorts [7]: we treated those cohorts as two separate studies. We found three additional relevant studies in the reference lists of retrieved articles [6, 14, 15]. Thus, including the present results from COSM, 12 prospective cohort studies were included in the meta-analysis. These studies included a total of 16,264 cases of type 2 diabetes documented in 287,963 men and women (Table 3). Five studies were conducted in the United States, two in Sweden, two in Finland, and one each in Spain, France and Japan.

**Table 3:** Characteristics of prospective studies of egg consumption and risk of type 2 diabetes

Study	Country; study name	Years of follow-up	Baseline examination	Cases (participants)	Category of egg intake	HR (95% CI)	Adjustments
Montonen et al, 2005 [14]	Finland; Finnish Mobile Clinic Health Examination Survey	23	1967–1972	383 (4,304 men and women aged 40–69 years)	<12 g/d	1.00 (reference)	Adjusted for age, sex, geographic area, BMI, smoking, family history of diabetes, total energy intake
					12–23 g/d	1.03 (0.79, 1.35)	
					24–40 g/d	0.89 (0.67, 1.18)	
					>40 g/d	0.91 (0.67, 1.23)	
Vang et al, 2008 [6]	USA; Adventist Mortality Study and Adventist Health Study	17	1960 in AMS and 1976 in AHS	543 (8,401 men and women aged 45–88 years)	Never	1.00 (Reference)	Age, sex
					>0 to <1/wk	1.32 (0.77, 2.25)	
					≥1/wk	1.15 (0.85, 1.54)	
Djoussé et al, 2009 [7]	USA; Physicians’ Health Study I	20	1982	1,921 (20,703 men aged ≥40 years)	0	1.00 (reference)	Age, BMI, vigorous exercise, smoking, history of hypercholesterolaemia and hypertension, alcohol intake
					<1 egg/wk	1.09 (0.87, 1.37)	
					1 egg/wk	1.09 (0.88, 1.34)	
					2–4 eggs/wk	1.18 (0.95, 1.45)	
					5–6 eggs/wk	1.46 (1.14, 1.86)	
					≥7 eggs/wk	1.58 (1.25, 2.01)	
Djoussé et al, 2009 [7]	USA; Women’s Health Study	11.7	1992–1995	2,112 (36,295 women aged ≥45 years)	0	1.00 (reference)	Age, BMI, exercise, smoking, family history of diabetes, history of hypertension and hypercholesterolaemia, intakes of energy, alcohol, red meat, fruits and vegetables, saturated fatty acids, *trans*-fatty acids, PUFAs
					<1 egg/wk	1.06 (0.92, 1.22)	
					1 egg/wk	0.97 (0.83, 1.12)	
					2–4 eggs/wk	1.19 (1.03, 1.38)	
					5–6 eggs/wk	1.18 (0.88, 1.58)	
					≥7 eggs/wk	1.77 (1.28, 2.43)	
Djoussé et al, 2010 [8]	USA; Cardiovascular Health Study	11.3	1989–1990 and 1992–1993	142 in men and 171 in women (1,669 men and 2,229 women aged ≥65 years)	Men		Age, race, field centre, BMI, physical activity, smoking, intakes of alcohol and cereal fibre
					Never	1.00 (reference)	
					<1/mth	0.95 (0.45, 2.01)	
					1–3/mth	1.14 (0.60, 2.15)	
					1–4/wk	0.96 (0.50, 1.82)	
					Almost daily	1.81 (0.77, 4.22)	
					Women		
					Never	1.00 (reference)	
					<1/mth	0.77 (0.43, 1.38)	
					1–3/mth	0.73 (0.47, 1.14)	
					1–4/wk	0.76 (0.47, 1.23)	
					Almost daily	0.38 (0.10, 1.37)	
Zazpe et al, 2013 [9]	Spain; SUN Project	6.6	1999–2008	91 (15,956 men and women aged 20–90 years)	<1 egg/wk	1.00 (reference)	Age, sex, BMI, leisure-time physical activity, smoking, family history of diabetes, hypertension, hypercholesterolemia, cardiovascular disease, Mediterranean food pattern, intakes of total energy and alcohol
					1 egg/wk	0.9 (0.4, 1.8)	
					2–4 eggs/wk	0.6 (0.3, 1.2)	
					>4 eggs/wk	0.7 (0.3, 1.7)	
Kurotani et al, 2014 [11]	Japan; Japan Public Health Center-based Prospective Study	5	1990 and 1993	672 in men and 493 in women (27,248 men and 36,218 women aged 45–75 years)	Men		Age, public health centre area, BMI, total physical activity, smoking, history of hypertension and family history of diabetes, intakes of total energy, alcohol, coffee, magnesium, calcium, rice, fish and shellfish, meat, vegetables, and soft drinks
					7.7 g/d (median)	1.00 (reference)	
					19.4 g/d	0.93 (0.74, 1.15)	
					32.6 g/d	0.93 (0.74, 1.16)	
					55.0 g/d	1.06 (0.85, 1.32)	
					Women		
					6.9 g/d (median)	1.00 (reference)	
					17.5 g/d	1.01 (0.79, 1.29)	
					29.4 g/d	0.94 (0.73, 1.21)	
					50.3 g/d	0.82 (0.63, 1.06)	
Virtanen et al, 2015 [13]	Finland; Kuopio Ischaemic Heart Disease Risk Factor Study	19.3	1984–1989	432 (2,332 men aged 42–60 years)	<14 g/d	1.00 (reference)	Age, examination year, education, family history of type 2 diabetes, BMI, leisure-time physical activity, smoking, hypertension, serum long-chain *n*-3 PUFAs, and intakes of total energy, alcohol, linoleic acid, fibre, fruit, berries, and vegetables
					14–26 g/d	0.91 (0.71, 1.18)	
					26–45 g/d	0.63 (0.48, 0.83)	
					>45 g/d	0.62 (0.47, 0.82)	
Djoussé et al, 2015 [12]	USA; Jackson Heart Study	7.3	2000–2004	531 (3,564 men and women aged 21–95 years)	<1/mth	1.00 (reference)	Age, sex, education, BMI, waist circumference, physical activity score, smoking, history of hypertension, history of cardiovascular disease, intakes of total energy, alcohol, red meat (including bacon), fruit and vegetables, fibre, magnesium, and *trans*-fatty acids
					1–3/mth	0.88 (0.65, 1.19)	
					1/wk	0.94 (0.68, 1.30)	
					2/wk	0.91 (0.66, 1.25)	
					3–4/wk	1.11 (0.81, 1.52)	
					≥5/wk	1.17 (0.81, 1.70)	
Ericson et al, 2015 [15]	Sweden; Malmö Diet and Cancer	14	1991–1996	2,797 (24,070 men and women aged 45–74 years)	4 g/d (median)	1.00 (reference)	Age, sex, method version, season, education, BMI, leisure-time physical activity, smoking, intakes of total energy and alcohol
					12 g/d	1.07 (0.95, 1.20)	
					19 g/d	0.99 (0.88, 1.12)	
					28 g/d	1.10 (0.98, 1.24)	
					45 g/d	1.14 (1.02, 1.28)	
Lajous et al, 2015 [10]	France; The E3N study	14	1993–1995	1,803 (65,364 women aged 43–70 years)	Never	1.00 (reference)	Age, education, BMI, smoking, physical activity, menopause, hormone replacement therapy, hypertension, hypercholesterolaemia, energy, alcohol, processed red meat, coffee, fruits, vegetables, sugar-sweetened and artificially sweetened drinks
					0.1–0.9 eggs/wk	0.84 (0.64, 1.11)	
					1–1.9 eggs/wk	0.91 (0.71, 1.17)	
					2–4.9 eggs/wk	0.94 (0.74, 1.20)	
					≥5 eggs/wk	1.00 (0.78, 1.29)	
Wallin et al, 2016 (current study)	Sweden; COSM	15	1997	4,173 (39,610 men aged 45–79 years)	<1/wk	1.00 (reference)	Age, education, BMI, physical activity, smoking, intakes of total energy, alcohol, coffee, red meat, processed meat, fish, fruits, vegetables, white bread, caviar, sweet buns/biscuits and fibre, and history of cardiovascular disease at baseline
					1–2/wk	0.98 (0.92, 1.05)	
					3–4/wk	1.11 (0.99, 1.24)	
					≥5/wk	1.11 (0.95, 1.29)	

AHS, Adventist Health Study; AMS, Adventist Mortality Study; d, day; mth, month; PUFA, polyunsaturated fatty acid; wk, week

**Fig. 1:**
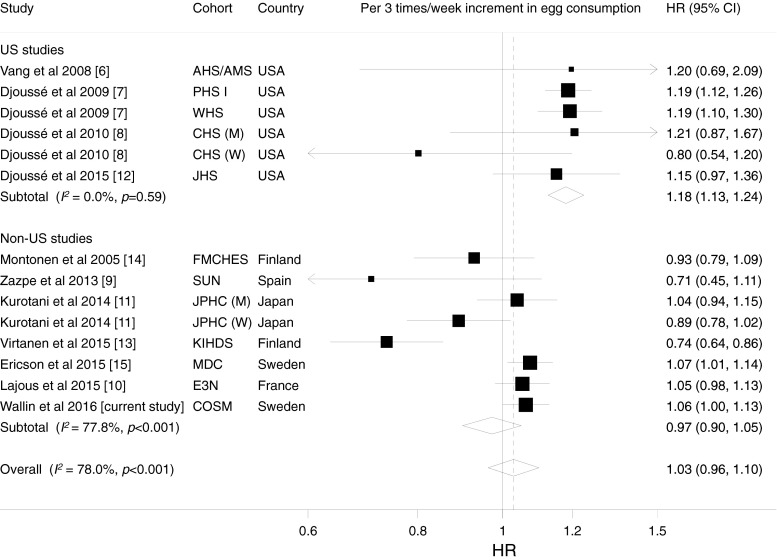
HRs for type 2 diabetes for each 3 times/week increment in egg consumption. HRs were combined using a random-effects model. Squares represent study-specific HR estimates (size of the square reflects the study-specific statistical weight); horizontal lines represent 95% CIs; diamonds represent the combined HRs with their 95% CIs. AHS, Adventist Health Study; AMS, Adventist Mortality Study; CHS, Cardiovascular Health Study; E3N, The E3N study; FMCHES, Finnish Mobile Clinic Health Examination Survey; JHS, Jackson Heart Study; JPHC, Japan Public Health Center-based Prospective Study; KIHDS, Kuopio Ischaemic Heart Disease Risk Factor Study; MDC, Malmö Diet and Cancer; PHS I, Physicians’ Health Study I; SUN, Sun Project; WHS, Women’s Health Study; M, men; W, women

There was no overall association between egg consumption and type 2 diabetes (HR for a 3 times/week increase in egg consumption 1.03 [95% CI 0.96, 1.10]), but there was significant heterogeneity among studies (*I*^2^ = 78.0%; *p* < 0.001). In a stratified analysis by sex, the overall HRs for every 3 times/week increment in egg consumption were 1.03 (95% CI 0.90, 1.17; *I*^2^ = 88.9%; *p* < 0.001) in men (five studies) and 1.02 (95% CI 0.89, 1.17; *I*^2^ = 80.6%; *p* = 0.001) in women (four studies). We observed substantial differences in association according to region, which partly explained the heterogeneity in the overall estimate (Fig. 1). Egg consumption was positively associated with type 2 diabetes risk in studies conducted in the USA, with no heterogeneity among studies (Fig. 1). In contrast, no overall association was observed in non-US studies; however, statistically significant heterogeneity remained among these studies (*I*^2^ = 77.8%; *p* < 0.001; Fig. 1). In a meta-regression analysis of non-US studies, we observed that the period of baseline data collection (before or after 1990; *p* = 0.05) but not sex, country or years of follow-up (*p* > 0.45 for all) was a potential source of heterogeneity. Among the non-US studies, the HRs for type 2 diabetes were 0.83 (95% CI 0.66, 1.03; *I*^2^ = 75.8%; *p* = 0.04) in the two studies (both conducted in Finland) with baseline data collection before 1990 and 1.04 (95% CI 0.98, 1.09; *I*^2^ = 46.5%; *p* = 0.10) in the five studies with baseline data collection after 1990. We found no evidence of a non-linear association between egg consumption and risk of type 2 diabetes in any study (*p* for non-linearity ≥0.15 for all). There was no evidence of publication bias (Egger’s test: *p* = 0.13 in the dose–response meta-analysis; *p* = 0.55 when comparing the highest and lowest categories of egg consumption).

## Discussion

In this large prospective cohort study of men, there was no significant association between egg consumption and risk of type 2 diabetes. In a meta-analysis of prospective cohort studies, a positive association between egg consumption and risk of type 2 diabetes was observed in US studies only, whereas no association was observed in studies conducted in European and Asian populations.

Eggs are nutrient dense and a good source of many important vitamins, minerals and carotenoids. However, they are also the major source of dietary cholesterol [28]. Although the overall fat composition of the diet is a more important determinant of blood cholesterol levels, dietary cholesterol may raise blood cholesterol somewhat [29]. Some [2, 3, 7, 30], but not all [11–13], cohort studies have reported a positive association between dietary cholesterol and risk of type 2 diabetes. Eggs are also rich in protein. A high intake of dietary protein [4, 5, 31], especially from animal sources [4, 30, 31], has been associated with higher risk of type 2 diabetes in several cohort studies, but not in all [32–34]. In the present study of Swedish men, the additional inclusion of dietary cholesterol or dietary protein intake in the multivariable model did not alter results. Randomised trials of the effects of high-egg diets (2–3 eggs/day for 6–12 weeks) on markers of glycaemic control and insulin resistance have generally found no change in fasting plasma glucose levels [35–38], the postprandial response to glucose [37], HbA_1c_ [35, 37] or fasting insulin levels [37]. However, one trial showed that a high-egg diet reduced plasma insulin levels and insulin resistance [36]. Thus, egg consumption seems to have either a null or beneficial effect on insulin sensitivity, and would therefore not be expected to elevate the risk of type 2 diabetes.

Egg consumption may be a marker of a broader dietary pattern associated with type 2 diabetes risk. Such food consumption habits could potentially vary between populations and may partly explain discrepancies between the reported results of different countries. In fact, in the Physicians’ Healthy Study I (one of the US studies that observed a significant positive association between egg consumption and type 2 diabetes risk [7]), egg consumption was related to the Western dietary pattern, characterised by a high consumption of red and processed meat, French fries, potatoes, high-fat dairy products, butter, snacks, sweets and desserts, and refined grains [39]. In contrast, the Japan Public Health Center-based Prospective Study found no association between egg consumption and type 2 diabetes [11]. In this study, egg consumption was more strongly associated with prudent, traditional dietary patterns than with the Western dietary pattern [40]. Available evidence indicates that unprocessed and processed red meat consumption, which is associated with the Western dietary pattern, is positively associated with risk of type 2 diabetes [41]. Although several studies have adjusted for red and/or processed meat consumption [7, 11, 12] or stated that additional adjustment did not substantially influence the risk estimates [8, 13, 15], residual confounding by meat or other foods potentially associated with weight gain and risk of type 2 diabetes (such as high-fat foods, snacks, sweets and refined grains) may have affected the results. In the COSM, egg consumption was positively associated with type 2 diabetes before adjustment for other dietary factors, but the association was markedly attenuated and lost after comprehensive adjustment for other components in the diet.

The strengths of our study include its prospective population-based design, the inclusion of detailed information on diet and other lifestyle factors, and the large number of incident cases. In addition, the use of national registers enabled objective ascertainment of type 2 diabetes cases and ensured comprehensive follow-up (not relying on response rates). However, owing to the progressive nature of the disease (with no clear onset) and the fact that it rarely leads to hospitalisation in the early stages, it is likely that some new cases are unregistered and were therefore missed. A limitation in the assessment of egg consumption is the lack of information on cooking methods and on consumption of egg white, egg yolk or both. Another limitation is that egg consumption as well as other dietary factors and covariates were assessed with a self-administered questionnaire at a single time point, which inevitably led to a degree of misclassification. Moreover, the observational design means that we cannot exclude the possibility that our findings may be influenced by unmeasured or residual confounding. Finally, as our study only included Swedish men (predominantly white), our results may not be generalisable to women or other ethnic groups.

One strength of this meta-analysis is the large sample size that provided high statistical power to detect weak associations that may have been missed in individual studies. The inclusion of prospective studies only prevented recall and selection bias, which could be a problem in retrospective studies. However, other limitations of the included studies were inherited by the meta-analysis. For example, misclassification or confounding in the original studies might have biased the results toward over- or underestimation of the risk estimates. Most studies had adjusted for age, sex, BMI, physical activity and smoking, with variable adjustment for other potentially confounding factors, especially other dietary factors. Another limitation is the lack of detailed information on egg consumption in most of the included studies (e.g. cooking methods and consumption of white and/or yolk). Differences in such factors might explain differences in results across studies. In addition, in most studies the extent to which the dietary assessment captured eggs cooked into dishes (omelettes, baked products etc.) was unclear. In meta-analyses, publication bias can lead to the overestimation of associations because studies with null results or small sample sizes tend not to be published. However, we found no evidence of publication bias in this meta-analysis.

In conclusion, the present findings from the prospective COSM do not support an association between egg consumption and risk of type 2 diabetes. In an updated meta-analysis of prospective studies, frequent egg consumption was associated with a higher risk of type 2 diabetes in US studies only. Egg consumption habits and associated overall dietary patterns may differ between populations and could potentially explain discrepancies between the reported results. Given the inconsistent findings across populations, the potential association between egg consumption and type 2 diabetes risk warrants further study in other large studies.

## Abbreviations

COSM: Cohort of Swedish Men
CVD: Cardiovascular disease
FFQ: Food frequency questionnaire
MET: Metabolic equivalent
